# Community-dwelling older people with an injurious fall are likely to sustain new injurious falls within 5 years - a prospective long-term follow-up study

**DOI:** 10.1186/1471-2318-14-120

**Published:** 2014-11-18

**Authors:** Petra Pohl, Ellinor Nordin, Anders Lundquist, Ulrica Bergström, Lillemor Lundin-Olsson

**Affiliations:** Department of Community Medicine and Rehabilitation, Physiotherapy, Umea University, 90187 Umea, Sweden; Department of Statistics, Umea School of Business and Economics, 90187 Umea, Sweden; Department of Surgical and Perioperative Science, Orthopedics, Umea University, 90187 Umea, Sweden

**Keywords:** Accidental falls, Older adults, Risk factors, Community-dwelling, Fall prediction

## Abstract

**Background:**

Fall-related injuries in older people are a leading cause of morbidity and mortality. Self-reported fall events in the last year is often used to estimate fall risk in older people. However, it remains to be investigated if the fall frequency and the consequences of the falls have an impact on the risk for subsequent injurious falls in the long term. The objective of this study was to investigate if a history of one single non-injurious fall, at least two non-injurious falls, or at least one injurious fall within 12 months increases the risk of sustaining future injurious falls.

**Methods:**

Community-dwelling individuals 75–93 years of age (*n* = 230) were initially followed prospectively with monthly calendars reporting falls over a period of 12 months. The participants were classified into four groups based on the number and type of falls (0, 1, ≥2 non-injurious falls, and ≥1 injurious fall severe enough to cause a visit to a hospital emergency department). The participants were then followed for several years (mean time 5.0 years ±1.1) regarding injurious falls requiring a visit to the emergency department. The Andersen–Gill method of Cox regression for multiple events was used to estimate the risk of injurious falls.

**Results:**

During the long-term follow-up period, thirty per cent of the participants suffered from at least one injurious fall. Those with a self-reported history of at least one injurious fall during the initial 12 months follow-up period showed a significantly higher risk for sustaining subsequent injurious falls in the long term (hazard ratio 2.78; 95% CI, 1.40–5.50) compared to those with no falls. No other group showed an increased risk.

**Conclusions:**

In community-dwelling people over 75 years of age, a history of at least one self-reported injurious fall severe enough to cause a visit to the emergency department within a period of 12 months implies an increased risk of sustaining future injurious falls. Our results support the recommendations to offer a multifactorial fall-risk assessment coupled with adequate interventions to community-dwelling people over 75 years who present to the ED due to an injurious fall.

## Background

Falls are a major health concern among older adults, and fall-related injuries are a leading cause of morbidity and mortality [1]. About 10% of all fall events result in serious injuries such as fractures or subdural hematomas, and falls account for more than 15% of visits to a hospital emergency department (ED) [2–4]. The risk of falling and sustaining fall-related injuries increases exponentially with age [1, 5–8]. After the age of 80 years 50% of community-dwelling people are expected to experience at least one fall each year [6, 9, 10]. Older people presenting to the ED for any reason are more likely to sustain fall-related injuries the following six months after discharge [11]. Older women are generally believed to be more likely to sustain non-fatal fall-related injuries than men [7, 12], but in very old people (older than 85 years) men have a greater risk of fall-related mortality compared to women [13, 14]. The costs involved with injurious falls are substantial, and may have the greatest consequences for people’s health [15], including increased risk of placement in long-term care facilities [3, 16].

In order to prevent and reduce fall-risk in older people it is important to identify individuals at risk of falling. A history of falling has been identified as one of the strongest independent risk factors for additional falls. Older people who have fallen within the past year are more likely to fall again [6, 17, 18], especially if they were hospitalized due to the fall [19]. The risk of falling is generally higher after recurrent falls, defined as two or more falls, than after one fall for community-dwelling people over 65 years [17, 20]. Furthermore, seeking emergency medical care because of a fall-related injury (no fracture) for the first time has been shown to be associated with an increased risk of later falls and fractures [12]. Based on this knowledge it has been recommended to ask all patients from 65 years of age in clinical settings about falls over the last 12 months. If the patient reports recurrent falls, or has been seeking medical attention because of an injurious fall, this should be considered as high-risk for renewed falls and a further evaluation, treatment, and referral is warranted. A fall-risk assessment is not considered necessary for people reporting only a single fall, if there are no reported or demonstrated difficulty or unsteadiness [21].

It has been questioned if one single non-injurious fall within 12 months should be seen as an elevated risk of renewed falls in older community-dwelling people, but instead be equated with no falls [22, 23]. This perspective has become increasingly common in prospective studies on falls. Proponents for this opinion argue that recurrent falls might indicate an underlying high-risk state that predisposes to falling, and that one fall might happen by chance alone [17, 24, 25]. The predisposing factors would be those related to various cumulated effects of chronic diseases and physiologic decline which may become more pronounced with age [26].

Even though the association between a history of falls and future falls has been well established, there is little known about the implications of the fall frequency and injurious falls for the event of subsequent injurious falls in the future. Observational data suggest that the risk factors for falls and for serious fall-related injuries are similar [2, 3]. It may therefore be assumed that previous falls also increase the risk of future injurious falls. In addition, since the risk of falling and sustaining fall-related injuries increase exponentially with age, it may be expected that community-dwelling people over 75 years of age with recurrent falls or who have been seeking medical attention due to an injurious fall, are more likely to sustain future injurious falls.

The objective of this study was to determine if a single non-injurious fall, recurrent non-injurious falls, or an injurious fall within a period of 12 months are associated with an increased long-term risk of experiencing injurious falls in community-dwelling people over 75 years of age.

## Methods

### Participants

A total of 230 community-dwelling people (64 men and 166 women) from Umeå, Sweden, between the ages of 75 years and 93 years (mean 79.5 years ±3.7 years) were recruited through senior citizen organisations, physiotherapists, occupational therapists in primary care, and advertisements in the local press between October 2004 and December 2005 [27]. Inclusion criteria were ≥75 years of age, the ability to walk at least 10 meters without a walking aid, and a cognitive function of 24 points or more on the Mini-Mental State Examination [28] in order to be able to follow instructions regarding follow-up on falls. The study was approved by the Regional Ethical Review Board in Umeå (Dnr 2011-191-31 M and 04-071 M), and all participants gave their written consent.

### Data collection

This study is based on a cross-sectional baseline assessment and two longitudinal data collections from the same sample of participants: 1) a detailed one-year follow-up on self-reported falls and fall-related injuries, and 2) a long-term follow-up on registered injurious falls on individuals presenting to the ED, including fall events that occurred during hospital admission.

#### Cross-sectional baseline assessment

The baseline assessment of the 230 participants included self-reported medical conditions and socio-demographic indicators regarding age, marital status, years of education, history of falls (previous 12 months) and fractures (previous five years), fear of falling, and medication use. The Barthel Index score of activities of daily living questionnaire [29] and the 15-item Geriatric Depression Scale [30] were filled out. Performance-based tests were completed, including the Short Physical Performance Battery (SPPB) [31] and preferred walking speed [32] using GAITRite® [33], an instrumented walkway system. We used a 10 meter walkway of which the middle 6.1 meters was registered by the GAITRite® system in order to minimize the effect of the acceleration and deceleration phase of the gait.

#### One-year follow-up on self-reported falls and fall-related injuries

Participants were prospectively followed for one year with monthly fall calendars. If the calendar was not returned on time, the participant was contacted by telephone. Whenever a fall was reported, the participants was contacted by telephone in order to gather information about the consequences and circumstances surrounding the fall. A fall was defined as an event in which the participant unintentionally came to rest on the floor or ground regardless of the cause or the consequences of the fall, and a fall-related injury as one that was severe enough to cause a visit at the ED. Both falls and fall-related injurious were self-reported by the participant. The rate of falls and observation time were recorded from the day of inclusion until voluntary dropout, death or the end of the follow-up period 365 days later [34].

#### Long term follow-up on registered injurious falls

Data from the 230 participants were matched with data from the Umeå University Hospital’s on-going injury registration – the Umeå Injury Database (IDB) – a data set of injuries due to accidents and trauma from the well-defined geographic area of Umeå. When visiting the ED at Umeå University Hospital, the injured, or an accompanying person filled out a questionnaire describing the situation causing the injury. When needed, the data set was supplemented with data from ambulance and police records, as well as medical records. Injury severity for up to three injuries per patient and event was registered. Data were registered in the database by personnel from the hospital’s accident surveillance group. For each event, data included information on gender, date, mechanism of and activity at time of injury, injury type, and treatment of injuries. The IDB is annually cross-checked with the general hospital register, and all falls causing severe injuries that occurred at the hospital were, therefore, included [35]. Dates of death were obtained from the Swedish Tax Agency’s register. Death more than three months after the injury event was not considered to be a direct consequence of the fall event. Causes of death were not investigated.

### Statistical methods

Data are reported as rates and proportions and as mean values ± standard deviations. The total observation time during the initial follow-up period was counted as the number of days at risk for falls and the incidence rate of injurious falls was presented as the number of falls per 100 person years (PY). The time to the registered injury event was calculated as the time from inclusion in the long-term follow-up until censoring or any event. An event was defined as a fall requiring a visit to the ED, and participants were censored at the end of the follow-up or at death. In order to reflect decision-making in clinical settings the starting time for the long-term follow-up differed for the groups. Those with no self-reported falls were included at their date of inclusion in the study, those with one self-reported non-injurious fall were included at the date of the actual fall, those with at least two self-reported non-injurious falls were included at the date of the second fall, and those with one self-reported injurious fall causing a visit to the ED were included at the date of the actual injurious fall. The time to the registered injury events were analysed using a Cox proportional hazards model, employing the Andersen–Gill extension to allow for multiple events per subject [36]. The independent variable of primary interest was self-reported fall categories based on the initial follow-up year. Potential confounders were included as covariates in the model: age, gender, SPPB score, and use of potential risk medications at baseline. The assumption of proportional hazards was tested for each individual covariate using Schoenfeld residuals [37]. No variable violated the proportional hazards assumption.

In order to assess the robustness of our findings we performed a sensitivity analysis [38] including all follow-up time for all groups within the first year, i.e. established the categories at the end of the first year and included the whole year. Analyses were performed using Stata (version 12, StataCorp, College Station, TX, USA). Results were considered significant if the associated *p*-value was below 0.05.

## Results

During the one-year prospective monitoring period of falls, 320 self-reported incidents were recorded based on the fall calendars, corresponding to an incidence rate of 95 falls/100 PY. One hundred eleven of the 230 participants (48%) fell at least once, and 54 (23%) fell at least twice according to the self-report fall calendar. There was no difference between women and men. Based on number of and severity of their self-reported falls during the monitoring period, participants were classified as *no falls* (*n =* 119; 52%); *one fall without injuries* (severe enough to cause a visit to the ED) (*n* = 51; 22%); *two or more falls without injuries* (*n* = 40; 17%); or *one injurious fall* (severe enough to cause a visit to the ED) (*n* = 20; 9%). Table 1 provides characteristics of the four groups.

**Table 1:** **Characteristics of participants with respect to falls during initial monitoring period and long-term follow-up**

	Total (***n***=230)	No falls (***n***=119)	1 fall, no injuries (***n***=51)	≥2 falls, no injuries (***n***=40)	≥1 fall, injuries (***n***=20)
Age, mean (SD)	79.5 (3.7)	79.0 (3.0)	80.0 (3.9)	79.0 (3.9)	82.8 (4.7)
Women, *n* (%)	166 (72)	89 (75)	38 (75)	22 (55)	17 (85)
Mini-Mental State Examination, score, mean (SD)	27.7 (1.8)	27.7 (1.8)	28.0 (1.7)	27.7 (1.9)	26.9 (2.2)
Living alone, *n* (%)	123 (54)	63 (53)	30 (59)	17 (43)	13 (65)
Use of walking aid indoors, *n* (%)	20 (9)	10 (8)	4 (8)	2 (5)	4 (20)
Fear of falling, *n* (%)	113 (49)	57 (48)	26 (51)	16 (40)	14 (70)
15-item Geriatric Depression Scale, 0–15 points, mean (SD)	1.7 (2.0)	1.6 (1.9)	1.4 (1.4)	1.5 (1.6)	3.6 (3.2)
**Diagnosis and use of drugs**					
Diabetes mellitus, *n* (%)	20 (9)	11 (9)	7 (14)	2 (5)	3 (15)
Previous stroke, *n* (%)	28 (12)	13 (11)	7 (14)	5 (13)	0 (0)
Heart disease, *n* (%)	53 (23)	27 (23)	10 (20)	12 (30)	4 (20)
Rheumatism/Arthritis, *n* (%)	15 (7)	7 (6)	4 (8)	3 (8)	1 (5)
Prescription drugs^a^ ≥1, *n* (%)	104 (45)	51 (43)	19 (37)	21 (52)	13 (68)
**Measures of Function**					
Barthel Index score, mean (SD)	19.9 (0.5)	19.8 (0.5)	19.9 (0.3)	19.9 (0.5)	19.7 (0.6)
Preferred gait speed (m/s) over a distance of 6.1 m, mean (SD)	1.1 (0.3)	1.1 (0.3)	1.1 (0.3)	1.1 (0.2)	0.9 (0.2)
Short Physical Performance Battery score, mean (SD)	10.1 (2.1)	10.3 (2.1)	10.0 (2.3)	10.6 (1.2)	8.8 (2.6)
**Previous fall before baseline** ^b^					
Single fall in previous year, *n* (%)	81 (35)	45 (38)	18 (35)	14 (35)	4 (20)
≥2 falls in previous year, *n* (%)	45 (20)	10 (8)	11 (22)	16 (40)	8 (40)
Fracture previous 5 years, *n* (%)	95 (41)	46 (39)	22 (43)	13 (25)	14 (70)
**Long-term follow-up**					
Individuals visiting the emergency department ≥1 time due to injurious falls, *n* (%)	70 (30)	33 (28)	16 (31)	12 (30)	9 (45)
Number of visits to the emergency department during long-term follow-up	91	39	18	15	19
Years of follow-up in the long-term follow-up, mean (SD)	5.0 (1.1)	5.3 (1.0)	5.0 (0.9)	4.6 (1.5)	4.9 (0.9)

^a^Potential risk medications include calcium preparations, potassium-sparing diuretics, oxicams, anilides, anxiolytics and hypnotics (both benzodiazepine derivatives).

^b^Participants were asked about falls that occurred within one year prior to inclusion and about fractures that occurred within five years prior to inclusion.

During the long-term follow-up, the total observation time was 1159 PY, the mean follow-up time was 5.0 (±1.1) years per participant (Table 1). Seventy individuals (30%) were registered in the ED for 91 unintentional injurious falls corresponding to an incidence rate of 7.9 injurious falls per 100 PY. No difference was found between women and men: of the 91 events, women accounted for 72 (involving 30% of all women), and men accounted for 19 (involving 23% of all men) (*p* = 0.200). Fifteen participants (7%) were registered in the ED on at least two occasions, four participants were registered three times, and one participant was registered four times for injurious falls. An event could result in more than one injury. Fractures, contusions, abrasions, and lacerations comprised more than 82% of injury diagnoses (Table 2). Fractures accounted for 28 (39%) of women’s and 4 (21%) of men’s injuries. During the long-term follow-up, 27 individuals (11%) died – 12 (19%) men and 15 (9%) women. No participant died within three months following an injurious fall.

**Table 2:** **Distribution of the most severe fall-related injuries**

Injury diagnosis	Total number of injuries (***n***= 91)	Injuries among men (***n***= 19)	Injuries among women (***n***= 72)
Concussion, n (%)	2 (2.2)	1 (5.3)	1 (1.4)
Internal injury, n (%)	2 (2.2)	0 (0.0)	2 (2.8)
Fracture, hip, n (%)	7 (7.7)	0 (0.0)	7 (9.7)
Fracture, other, n (%)	25 (27.5)	4 (21.1)	21 (29.2)
Laceration, n (%)	16 (17.6)	6 (31.6)	10 (13.9)
Contusion/bruise, n (%)	26 (28.6)	4 (21.1)	22 (30.5)
Abrasion, n (%)	1 (1.1)	1 (5.3)	0 (0.0)
Luxation, n (%)	4 (4.4)	1 (5.3)	3 (4.2)
Strain/sprain, n (%)	3 (3.3)	0 (0.0)	3 (4.2)
Other, n (%)	5 (5.5)	2 (10.3)	3 (4.2)

**Figure 1:**
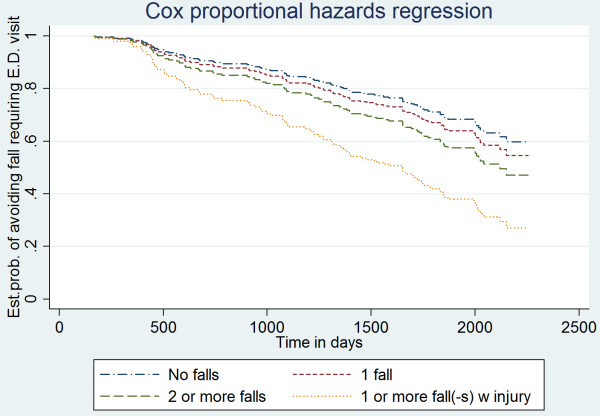
**Estimated probability of avoiding injurious falls requiring a visit to the emergency department.**

Analyses of the long-term follow-up of registered injurious falls showed that the group with at least one self-reported injurious fall during the initial monitoring 12 months showed a significantly higher risk for sustaining subsequent injurious falls severe enough to cause a visit to the ED in the long term (hazard ratio 2.78; 95% CI, 1.40–5.50) compared to those with no falls (Table 3 and Figure 1). There was no significant difference between the groups with no falls, one fall or at least two falls regarding risk of injurious falls in the long term. None of the potential confounders were significantly related to the outcome based on self-reported falls during the follow-up year.

**Table 3:** **Hazard ratio for injurious falls in long term in four defined categories**

Fall category	Unadjusted HR (95% CI)	***p***-value	Adjusted HR (95% CI)	***p***-value
No falls (*n* = 119)	1 (reference)		1 (reference)	
One single fall without injury (*n* = 51)	1.23 (0.72–2.10)	0.45	1.17 (0.69–1.98)	0.55
At least two non-injurious falls (*n* = 40)	1.41 (0.77–2.61)	0.27	1.51 (0.79–2.88)	0.22
One injurious fall (*n* = 20)	3.38 (1.73–6.62)	< 0.001	2.78 (1.40–5.50)	0.003

Adjusted for age, gender, Short Physical Performance Battery score, and potential risk medications.

The sensitivity analysis confirmed that the results remain robust as the results were similar to those based on primary analysis (hazard ratio 2.32; 95% CI, 1.15 – 4.68 for the group with injurious falls, only).

## Discussion

Our findings indicate that community-dwelling women and men over 75 years of age who sustain at least one injurious fall, severe enough to cause a visit to the ED, have an almost threefold risk of experiencing further injurious falls within the next five years. The findings are in line with previous research showing that older people presenting to the ED due to an injurious fall, especially a fracture, have a 50% higher risk to post-discharge injurious falls the forthcoming year [4, 8]. It should be noted that a substantial number, almost one third, of those with no, a single, or recurrent falls during the initial monitoring year also experienced injurious falls during the long-term follow-up which were severe enough to seek care at the hospital ED. However, our results did not significantly differ between these groups regarding the risk of sustaining a future injurious fall. In contrast to large population-based studies of older people presenting to the ED due to injurious falls [7, 12] we found no significant difference between women and men in the incidence rate of injurious falls, with an exception for the fracture rate: a larger proportion of women sustained a fracture.

Our results support previous recommendations that community-dwelling people over 75 years who present to the ED due to an injurious fall should be offered a multifactorial fall-risk assessment coupled with adequate interventions [8, 21]. Not all older people with fall-related injuries arrive at the ED [39], but the modifiable fall-risk factors have been found to be similar whether they visit the ED or not [40]. There are many recommendations and algorithms available on how to identify older people at high risk of falls [21, 41]. However, the recommendation to annually reassess every person from the age of 65 years in clinical settings who report one single non-injurious fall, and subsequently counselling them about taking part in fall-prevention exercise groups [21], may be very resource consuming for public health services. It may also be seen as a violation of the person’s autonomy if the patient seeks health care for reasons other than balance, gait disturbances or injurious falls. That said, since there is now convincing evidence that both the number of people that fall and the fall rate can be reduced in community-dwelling people over 65 years, regardless of risk factors [42, 43], older people should be encouraged to participate in public health initiatives offering the general benefits of strength and balance training and health services should make arrangements for such activities to be available at home or in groups.

Recurrent falls and high age have frequently been found to be significant risk factors for future injurious falls [1, 3, 5–7]. Our findings do not support the view that independent community-dwelling people over 75 years who have non-injurious recurrent falls the previous year have a higher risk of sustaining future injurious falls. However, if there are injuries involved with the recurrent falls there is reason to perform a systematic multifactorial fall-risk assessment to identify possible underlying pre-disposing factors. Regarding age, the risk of sustaining fall-related injuries has been found to increase steeply with increasing age. A large population-based study found that the rates of injurious falls for adults 85 years and older were four to five times that of adults aged 65–69 years [7]. It has been argued that it is not age *per se* that causes traumatic falls, but rather pre-existing accumulation of other risk factors as people age [13]. Increasing age may lead to changes in vision, postural control, slowed protective reflexes, muscle strength, and step height, which may impair older people’s ability to avoid a fall after an unexpected trip or while reaching or bending [8]. In our study, we analyzed age as a potential confounder to injurious falls but found no significant relationship.

Our study has limitations and strengths. There is a potential selection bias in using a volunteer sample with no cognitive impairments. This might not accurately represent the independent community-dwelling population at large. However, the results from the one-year follow-up are in agreement with population-based studies showing that about 25% of people over 75 years fall at least twice every year [3, 22], although lower and higher rates have also been found [44, 45]. The proportion of falls requiring medical care is in agreement with others [41], and the distribution of injury types during the long-term follow-up also corresponds well with other studies [7], and therefore we suggest that our sample represents the population regarding falls in this age group well. It is however important to note that we only included community-dwelling people at baseline and hence, the results may not be applicable to all older people. One strength is the detailed data collection of self-reported falls during the initial monitoring year and the thorough data collection of registered injurious falls at the ED for a longer period based on the procedures of the Umeå Injury Database. Recollection bias is a common source of misinterpretation because older people may forget that they have fallen. We have followed the recommendations for collecting self-reported data on falls with monthly reports [46] and thus the risk of recollection bias can be considered as low. In addition, we ensured that the individuals were cognitively intact prior to the study. We have used an extended form of Cox regression model allowing for multiple events, which is an additional strength. Furthermore, we performed a sensitivity analysis to confirm the robustness of our results.

## Conclusion

Community-dwelling people over 75 years of age with a history of at least one injurious fall severe enough to present to the ED within a period of 12 months implies an increased risk of sustaining injurious falls in the future. Our results support the recommendations to offer a multifactorial fall-risk assessment coupled with adequate interventions to community-dwelling people over 75 years who present to the ED due to an injurious fall.

## Abbreviations

ED: Emergency department
AGS/BGS: The American Geriatric Society and the British Geriatric Society
SPPB: Short physical performance battery
IDB: Injury data base
PY: Person years
SD: Standard deviation
HR: Hazard ratio
CI: Confidence interval.
